# Laparoscopy in Pregnancy: A Comparative Review of National Guidelines

**DOI:** 10.7759/cureus.38904

**Published:** 2023-05-11

**Authors:** Georgios Michos, Themistoklis Dagklis, Evangelos Papanikolaou, Nikolaos I Peitsidis, Ioannis A Kalogiannidis, Apostolos M Mamopoulos, Apostolos Athanasiadis

**Affiliations:** 1 Third Department of Obstetrics and Gynecology, School of Medicine, Faculty of Health Sciences, Aristotle University of Thessaloniki, Thessaloniki, GRC; 2 Private IVF Unit, Assisting Nature Centre of Reproduction and Genetics, Thessaloniki, GRC

**Keywords:** investigation, societies, guidelines, pregnancy, laparoscopy

## Abstract

Gynecological and general surgical conditions requiring surgical management during pregnancy constitute a medical challenge, which often entails the collaboration of numerous medical specialties. In recent years, laparoscopy in pregnancy has been accepted as a safe alternative to open surgery. This has led gynecological societies to conduct studies and issue guidelines related to laparoscopy in pregnancy, with a view to assisting and guiding clinicians and surgeons.

The aim of this study was to review and compare the recommendations from various published national guidelines on laparoscopy in pregnant women. To that end, a descriptive review of guidelines from the British Society for Gynaecological Endoscopy (BSGE), the Society of American Gastrointestinal and Endoscopic Surgeons (SAGES), the Society of Obstetricians and Gynaecologists of Canada (SOCG), and the Collège National des Gynécologues et Obstétriciens Français (CNGOF) was conducted.

Regarding diagnosis, the SAGES and SOCG societies recommend ultrasound as the preferred and safe imaging technique during pregnancy. In terms of the optimal timing for laparoscopic intervention, BSGE and SAGES do not restrict the laparoscopic approach based on safety, depending on the gestation week, whereas SOCG and CNGOF propose early second trimester and first and second quarter of pregnancy respectively. There is an overall consensus regarding patient positioning, initial port placement, insufflation pressure during the operation, venous thromboembolic (VTE) prophylaxis, fetal heart monitoring, and tocolysis among the reviewed guidelines. Moreover, only the BSGE mentions the need for corticosteroids, magnesium sulfate, and anti-D prophylactic administration.

## Introduction and background

It is of the utmost importance for healthcare providers to be alert to surgical, non-obstetric conditions that can occur during pregnancy, weighing the benefits and costs for both mother and fetus. Medical conditions requiring surgery in gravid women are rare and occur in 1-2/1000 pregnancies [1]. The most common non-obstetric emergencies complicating pregnancy are appendicitis, cholecystitis, and complicated adnexal masses (rupture or torsion) [2-3]. The use of laparoscopy has grown over the past years in the treatment of many surgical conditions, and it is used in pregnancy as well, with comparable outcomes and safety with open surgery. However, laparoscopy presents a dilemma in pregnancy, firstly because of the risk of uterine injury from initial port placement and secondly owing to the possible fetal distress due to high intrabdominal pressure, as a result of the creation of pneumoperitoneum.

Several scientific societies have issued guidelines regarding laparoscopy in pregnancy, e.g., the British Society for Gynaecological Endoscopy (BSGE), the Society of American Gastrointestinal and Endoscopic Surgeons (SAGES), the Society of Obstetricians and Gynaecologists of Canada (SOCG), and the Collège National des Gynécologues et Obstétriciens Français (CNGOF). The American College of Obstetricians and Gynecologists (ACOG) published its opinion titled “Nonobstetric Surgery During Pregnancy” in 2019, without mentioning laparoscopy as an alternative to open surgery, and hence we excluded this committee's opinion from our study [4-8].

Technological advances in endoscopy and the availability of well-trained surgeons and advanced anesthesiologic modalities have made laparoscopy the preferred surgical technique in gravid patients. Moreover, laparoscopy has the advantage over open surgery as it is associated with faster recovery, shorter hospital stays, and a lower rate of wound infection [9].

Given the challenges of conducting large-scale randomized trials in pregnancy, especially due to the rarity of surgical conditions during this period, it has not been possible for physicians to infer standardized recommendations from specified data. Hence, there is still a lack of consistent international protocols to offer a common pathway to all practitioners and optimize fetal and maternal outcomes. The aim of this comparative review is to summarize the recommendations from four influential national medical societies regarding the practice of laparoscopy during pregnancy.

## Review

The most recently published guidelines on laparoscopy during pregnancy were retrieved and a descriptive review was conducted. In particular, guidelines from the following four national societies were identified: BSGE [1], SAGES [3], SOCG [4], and CNGOF [5]. An overview of the recommendations is presented in the tables below.

Diagnosis

Abdominal pain is a common symptom in pregnant patients and often poses a challenge where medical practitioners must weigh the risk and benefits of diagnostic approaches to both the mother and the fetus. Ultrasound is an easily applicable, safe, and low-cost technique that can help healthcare providers to establish a diagnosis. SAGES and SOCG agree that ultrasound is safe and can be effective in determining abdominal pathology; however, the method’s efficacy depends on the operator's skill and experience [10-11] (Table 1).

**Table 1:** Summary of the recommendations on laparoscopy in pregnancy RCOG: Royal College of Obstetricians and Gynaecologists; JOGC: Journal of Obstetrics and Gynaecology Canada

	British Society for Gynaecological Endoscopy (BSGE), endorsed by RCOG	Society of American Gastrointestinal and Endoscopic Surgeons (SAGES)	Society of Obstetricians and Gynaecologists of Canada (SOCG) (editorial in JOGC)	Collège National des Gynécologues et Obstétriciens Français (CNGOF)
Country	UK	USA	Canada	France
Year issued	2019	2016	2019	2011
Title	Evidence-Based Guideline on Laparoscopy in Pregnancy	Guidelines for the Use of Laparoscopy During Pregnancy	Laparoscopy in Pregnancy	Risks Associated With Laparoscopic Entry: Guidelines for Clinical Practice From the French College of Gynecologists and Obstetricians
Pages	21	31	2	8
References	91	227	3	86
Diagnosis	Not mentioned	Ultrasound is safe and effective; MRI without the use of intravenous gadolinium can be performed at any stage of pregnancy	Ultrasound and MRI if needed; CT only in emergency situations	Not mentioned

When, in some cases, sonography is not diagnostic, more thorough imaging of the abdomen or pelvis is required, and MRI can be safely performed at any stage of pregnancy, without exposing the patient and the developing fetus to ionizing radiation. However, the use of intravenous gadolinium is not recommended and must be reserved for cases where it is deemed absolutely essential. Finally, CT should not be the initial imaging test of choice, due to the risk of radiation exposure to the fetus, except in emergency situations when urgent information is required and other imaging modalities are inconclusive (SAGES) [12].

Trimester of pregnancy

As laparoscopy may be associated with maternal and neonatal morbidities, all guidelines consider the gestation age so that the operation can be safely performed. The size of the uterus, depending on the trimester of pregnancy, may increase the difficulty of the operation accordingly. BSGE and SAGES recommend that laparoscopy can be safely performed regardless of the trimester of pregnancy and should not be delayed when the procedure is deemed necessary [13]. These guidelines contrast with the traditional suggestions to avoid surgery during the first and third trimesters due to the increased risk of negative outcomes for the pregnancy or the fetus [14]. However, SOCG and CNGOF disagree with the aforementioned societies and propose that laparoscopy can be performed in the early second trimester and the first and second quarters of pregnancy, respectively (Table 2).

**Table 2:** Trimester of pregnancy and laparoscopic intervention RCOG: Royal College of Obstetricians and Gynaecologists; JOGC: Journal of Obstetrics and Gynaecology Canada

	British Society for Gynaecological Endoscopy (BSGE), endorsed by RCOG	Society of American Gastrointestinal and Endoscopic Surgeons (SAGES)	Society of Obstetricians and Gynaecologists of Canada (SOCG) (editorial in JOGC)	Collège National des Gynécologues et Obstétriciens Français (CNGOF)
Trimester of pregnancy	Any trimester of pregnancy	Any trimester of pregnancy	Early second trimester	First and second quarters of pregnancy

Patient positioning

Our review of the guidelines revealed that SAGES, SOCG, and CNGOF recommend the left lateral or left partial lateral decubitus position in women in advanced pregnancies as the optimal position. By adopting this position, the pressure of the enlarged uterus on the inferior vena cava is minimized, thereby increasing venal return and cardiac output [15-16] (Table 3).

**Table 3:** Patient positioning, port placement, insufflation pressure RCOG: Royal College of Obstetricians and Gynaecologists; JOGC: Journal of Obstetrics and Gynaecology Canada

	British Society for Gynaecological Endoscopy (BSGE), endorsed by RCOG	Society of American Gastrointestinal and Endoscopic Surgeons (SAGES)	Society of Obstetricians and Gynaecologists of Canada (SOCG) (editorial in JOGC)	Collège National des Gynécologues et Obstétriciens Français (CNGOF)
Patient positioning	Not mentioned	Patients beyond the first trimester should be placed in the left lateral or partial left lateral decubitus position	The left lateral recumbent position is recommended in advanced pregnancy	In the third trimester, a table inclined towards the patient's left side to minimize compression of the inferior vena cava
Initial port placement	Depends on the level of the uterine fundus	The surgeon’s preference; and location adjusted according to fundal height	Primary trocar 5 cm higher than the upper part of the uterus	Depends on the volume of the uterus
Insufflation pressure	20-25 mmHg before inserting primary trocar, operating pressures of 12 mmHg	10-15 mmHg (adjusted based on the patient's physiology)	Up to 12 mmHg	Maximum of 12 mmHg

Initial port placement

There is a consensus among all the reviewed guidelines that the location of the primary port varies and is dependent upon the level of the uterine fundus. In fact, SOCG specifies that the primary trocar must be inserted 5 cm higher than the upper part of the uterus, alternatively at Palmer’s point [17], while CNGOF proposes either the open technique (transumbilical or supraumbilical route, especially after 24 weeks of pregnancy) or micro-laparoscopy through Palmer’s point [18-19]. BSGE mentions that given the absence of randomized controlled trials (RCTs), it should be based on the operator’s experience, and the choice of primary port location including umbilical, sub/xiphoid, or Palmer’s point [20- 21] (Table 3).

Insufflation pressure

Comparing the current guidelines, there is a consistent strategy regarding the insufflation pressure during laparoscopic surgery in pregnancy. It is stated that after achieving pressures of 20-25 mmHg before inserting the primary trocar (BSGE), the accepted and safe operating pressures that surgeons should aim for are up to 12 mmHg. Only SAGES states that operating pressures should be based on each patient’s comorbidities and physiology, within the limits of 10-15 mm Hg [17,22-26] (Table 3).

Adnexal mass - appendicitis - cholecystitis

Apart from CNGOF, all the other guidelines deal with the management of adnexal masses in pregnancy. It is generally accepted that expectant management and post-partum treatment is the preferred option in gravid patients. The BSGE recommends that in symptomatic, large masses, without torsion, aspiration under ultrasound guidance may be offered to alleviate symptoms as an alternative option [27].

According to SAGES guidelines, a mass can be managed expectantly provided that the initial ultrasound is not suggestive of malignancy and the lesion is not larger than 6 cm [28-29]. All societies have noted that, if medically necessary, surgery should not be postponed and a laparoscopic approach is preferable, depending on the surgeon’s expertise (SOCG). Moreover, SAGES guidelines recommend the laparoscopic approach to diagnose and treat torted adnexal masses.

BSGE guidelines touch on the need for surgical management in cases of appendicitis and cholecystitis in pregnancy, as complications (peritonitis and maternal sepsis) can result in poor pregnancy outcomes (miscarriage, preterm delivery) [1]. Laparoscopy should be considered the surgical approach of choice and should be carried out by experienced laparoscopists (Table 4).

**Table 4:** Nonobstetric pathology RCOG: Royal College of Obstetricians and Gynaecologists; JOGC: Journal of Obstetrics and Gynaecology Canada; MRI: magnetic resonance imaging

	British Society for Gynaecological Endoscopy (BSGE), endorsed by RCOG	Society of American Gastrointestinal and Endoscopic Surgeons (SAGES)	Society of Obstetricians and Gynaecologists of Canada (SOCG) (editorial in JOGC)	Collège National des Gynécologues et Obstétriciens Français (CNGOF)
Appendicitis	Surgical intervention is preferable as delay is associated with adverse maternal and fetal complications	Laparoscopy is the treatment of choice. MRI reduces negative exploration rate by 50%	Not mentioned	Not mentioned
Cholecystitis	Laparoscopy does not lead to increased risk for mother and fetus	Laparoscopic cholecystectomy is the treatment of choice in pregnant patients with symptomatic gallbladder disease, regardless of trimester	Not mentioned	Not mentioned
Adnexal mass	The risk of torsion in pregnancy is low; large non-torted symptomatic cysts may be offered aspiration under ultrasound guidance	Observation is acceptable, provided ultrasound is not concerning for malignancy and lesion <6 cm in size. Laparoscopy is recommended for both diagnosis and treatment of adnexal torsion	Depending on the surgeon's expertise, laparoscopy is preferable	Not mentioned

Perioperative care

The BSGE, SAGES, and SOCG guidelines comment on the use of fetal heart monitoring in relation to laparoscopic surgery. There is an overall agreement that fetal monitoring before and after an operation is indicated to establish fetal well-being, contrary to the traditional belief that monitoring intraoperatively is more accurate in identifying fetal distress during laparoscopy (SAGES). Concurrent contraction monitoring to confirm the absence of contractions may also be implemented in fetuses considered viable (Table 5).

**Table 5:** Fetus monitoring RCOG: Royal College of Obstetricians and Gynaecologists; JOGC: Journal of Obstetrics and Gynaecology Canada

	British Society for Gynaecological Endoscopy (BSGE), endorsed by RCOG	Society of American Gastrointestinal and Endoscopic Surgeons (SAGES)	Society of Obstetricians and Gynaecologists of Canada (SOCG) (editorial in JOGC)	Collège National des Gynécologues et Obstétriciens Français (CNGOF)
Fetal heart monitoring	Βefore and after surgery to confirm fetal wellbeing	Pre and postoperatively in a fetus considered viable	Unclear whether continuous fetal monitoring is needed when operating on a woman with a viable fetus	Not mentioned

Regarding thromboprophylaxis, only the BSGE guidelines suggest that the added risk of clot formation in pregnant women undergoing laparoscopic surgery must be evaluated and, hence, patients should be screened and receive the appropriate perioperative treatment [23-24]. In contrast, the SAGES and SOCG recommendations only mention intra and postoperative pneumatic compression of the lower limbs, as well as rapid postoperative ambulation as prophylactic measures (SAGES) [26].

Regarding the use of tocolysis, the BSGE, SAGES, and SOCG advise against its use on a routine basis and it should be reserved for cases when signs of preterm labor are present. Finally, only BSGE discusses perioperative anti-D management in gravid women undergoing laparoscopy. It notes that laparoscopic surgery is not considered a sensitizing event and, thus, routine anti-D administration is not regarded as necessary [30].

Corticosteroid and magnesium sulfate use should be contemplated for patients with fetuses at viable premature gestational ages; however, in the case of septic patients, a risk-benefit discussion must be conducted, and caution is advised (BSGE) (Table 6).

**Table 6:** Perioperative medical management RCOG: Royal College of Obstetricians and Gynaecologists; JOGC: Journal of Obstetrics and Gynaecology Canada

	British Society for Gynaecological Endoscopy (BSGE), endorsed by RCOG	Society of American Gastrointestinal and Endoscopic Surgeons (SAGES)	Society of Obstetricians and Gynaecologists of Canada (SOCG) (editorial in JOGC)	Collège National des Gynécologues et Obstétriciens Français (CNGOF)
Tocolytics	Routine tocolysis is not recommended	Should not be used, but should be considered perioperatively when signs of preterm labor are present	No clear benefit of using tocolytic agents before, during, or after the operation	Not mentioned
Anti-D prophylaxis	Is not deemed necessary	Not mentioned	Not mentioned	Not mentioned
Corticosteroids and magnesium sulfate	Should be administered, depending on the gestation of the fetus; caution in maternal sepsis	Not mentioned	Not mentioned	Not mentioned
Venous thromboembolic (VTE) prophylaxis	VTE prophylaxis should be considered	Intraoperative and postoperative pneumatic compression devices and early postop ambulation are recommended	Intermittent pneumatic pressure of the lower limbs	Not mentioned

## Conclusions

The aim of our study was to review and compare all current recommendations from national and international published guidelines on laparoscopy during pregnancy. The main issue of debate is as follows: which trimester of pregnancy is the optimal time for intervention? SOCG and the CNGOF propose certain limitations depending on the week of gestation, while BSGE and SAGES recommend that it can be performed regardless of the trimester of pregnancy. There is an agreement among three out of four societies that insufflation pressure during operation should be as low as 12 mmHg. It is also agreed that pregnant patients operated on laparoscopically should be screened for the risk of thromboembolism and that fetal heart monitoring before and after the operation is sufficient for ensuring the fetus's well-being. Furthermore, the expectant management of adnexal masses in pregnancy is suggested by the BSGE and SAGES guidelines, provided that no concerning signs of malignancy are revealed in the initial workup. However, surgery should not be postponed when it is deemed medically necessary, i.e., torted masses.

Pregnancy is a specific condition where skepticism about the risk of harm to the embryo often leads to confusion in terms of clinical decisions when dealing with a possible surgical diagnosis. In this era of significant advances in laparoscopy and better training of surgeons, more surgical, non-obstetric conditions will be managed endoscopically during pregnancy. We believe that the summarized recommendations from all current guidelines will guide physicians when they face dilemmas related to issues such as the optimal trimester of surgical intervention and the risk of harm to the embryo or the enlarged uterus. On the other hand, the absence of recommendations from some committees will become more evident and it will hopefully urge them to address the various issues regarding laparoscopy in pregnancy.
